# *DRDB*: An Online Date Palm Genomic Resource Database

**DOI:** 10.3389/fpls.2017.01889

**Published:** 2017-11-02

**Authors:** Zilong He, Chengwei Zhang, Wanfei Liu, Qiang Lin, Ting Wei, Hasan A. Aljohi, Wei-Hua Chen, Songnian Hu

**Affiliations:** ^1^CAS Key Laboratory of Genome Sciences and Information, Beijing Institute of Genomics, Chinese Academy of Sciences, Beijing, China; ^2^College of Life Science and Technology, Huazhong University of Science and Technology, Wuhan, China; ^3^University of Chinese Academy of Sciences, Beijing, China; ^4^Grail Scientific Co., Ltd., Shenyang, China; ^5^Joint Center for Genomics Research, King Abdulaziz City for Science and Technology and Chinese Academy of Sciences, Riyadh, Saudi Arabia

**Keywords:** date palm, short sequence repeat, single nucleotide polymorphism, genome variation, cultivar classification

## Abstract

**Background:** Date palm (*Phoenix dactylifera* L.) is a cultivated woody plant with agricultural and economic importance in many countries around the world. With the advantages of next generation sequencing technologies, genome sequences for many date palm cultivars have been released recently. Short sequence repeat (SSR) and single nucleotide polymorphism (SNP) can be identified from these genomic data, and have been proven to be very useful biomarkers in plant genome analysis and breeding.

**Results:** Here, we first improved the date palm genome assembly using 130X of HiSeq data generated in our lab. Then 246,445 SSRs (214,901 SSRs and 31,544 compound SSRs) were annotated in this genome assembly; among the SSRs, mononucleotide SSRs (58.92%) were the most abundant, followed by di- (29.92%), tri- (8.14%), tetra- (2.47%), penta- (0.36%), and hexa-nucleotide SSRs (0.19%). The high-quality PCR primer pairs were designed for most (174,497; 70.81% out of total) SSRs. We also annotated 6,375,806 SNPs with raw read depth≥3 in 90% cultivars. To further reduce false positive SNPs, we only kept 5,572,650 (87.40% out of total) SNPs with at least 20% cultivars support for downstream analyses. The high-quality PCR primer pairs were also obtained for 4,177,778 (65.53%) SNPs. We reconstructed the phylogenetic relationships among the 62 cultivars using these variants and found that they can be divided into three clusters, namely North Africa, Egypt – Sudan, and Middle East – South Asian, with Egypt – Sudan being the admixture of North Africa and Middle East – South Asian cultivars; we further confirmed these clusters using principal component analysis. Moreover, 34,346 SSRs and 4,177,778 SNPs with PCR primers were assigned to shared cultivars for cultivar classification and diversity analysis. All these SSRs, SNPs and their classification are available in our database, and can be used for cultivar identification, comparison, and molecular breeding.

**Conclusion:** *DRDB* is a comprehensive genomic resource database of date palm. It can serve as a bioinformatics platform for date palm genomics, genetics, and molecular breeding. *DRDB* is freely available at http://drdb.big.ac.cn/home.

## Introduction

Date palm (*Phoenix dactylifera* L.) is a widely cultivated plant species with agricultural and economic importance in the world. It is a dioecious plant in palm family Arecaceae with long life cycle, long period of juvenility and various of cultivars (El Hadrami et al., 2011). As the most important crop, Saudi Arabia has an estimated 25 million date palms producing nearly a million tons of dates annually. More than 400 different date palm cultivars are reported to exist in Saudi Arabia (Anonymous, 2006).

Based on the next generation sequencing, date palm genome sequence has been released and many date palm cultivars have been re-sequenced recently (Al-Dous et al., 2011; Al-Mssallem et al., 2013; Hazzouri et al., 2015). Short sequence repeat (SSR) and single nucleotide polymorphism (SNP) are very useful in plant genome analysis and breeding. Billotte et al. (2004) developed the nuclear SSR markers and investigated the genetic diversity for date palm while Hamwieh et al. (2010) analyzed the SSRs across the whole date palm genome. Based on SNPs among 11 date palm varieties, Al-Mssallem et al. (2013) noticed that the northern African varieties are significantly diverged from the Middle Eastern varieties.

To better understand the genetic basis of different date palm cultivars, it is necessary to have a high-quality genome assembly and get insight into genome variations (SSRs and SNPs). Here, based on 130X HiSeq data produced by our lab, we first updated the date palm genome assembly. Then, we annotated SSRs and other types of sequence variants among 62 cultivars. To facilitate the use of these data, we developed a web-based date palm genome database (*DRDB*). We included in *DRDB* in total of 6,375,806 SNPs and 246,445 SSRs from 62 cultivars. The main purpose of *DRDB* is to help the researchers/breeders to distinguish the plethora of date palm cultivars by using well-selected polymorphic markers (SNPs or SSRs). With *DRDB*, users can search for SNPs or SSRs of interests, and easily find SNP/SSR markers that are unique to user-selected cultivars. In addition, we also provided detailed annotations for the SNPs and SSRs. With *DRDB*, we not only expanded and enhanced the date palm cultivars data contents, but also incorporated useful tools to mine the data. To our knowledge, *DRDB* is the first comprehensive genetic variation database for date palm.

## Materials and Methods

### Plant Materials

Fresh green leaves from an adult date palm plant of *Khalas* (female) cultivar in Al-Hssa Oasis (24^∘∘^08′54′′N, 47°18′18′′E) were collected, washed with double distilled water, and frozen immediately in a liquid nitrogen container. After transported to the laboratory, these samples were stored in -80°C freezers until use.

### Genomic DNA Isolation and Sequencing

Genomic DNA was isolated from 50 g fresh leaves according to a CTAB-based method as before (Al-Mssallem et al., 2013). Briefly, 5 mg purified DNA was used for constructing the Illumina HiSeq libraries. HiSeq paired-end (180, 300, and 500 bp) and mate-pair libraries (1, 3, 5, and 8 kb) were constructed using the Illumina Simple Paired-End Library and Mate-Pair Library Preparation Protocol, respectively. The libraries were sequenced by Illumina HiSeq 2000 platform.

### Genome Assembly

We obtained ∼130X HiSeq data using Illumina HiSeq 2000 platform. The raw data was filtered using an in-house Perl script firstly. Then, SOAPdenovo2 (v2.04) was used to build a new genome assembly based on the newly produced HiSeq data with the option “-k 63” and other default parameters (Luo et al., 2012). Next, the new genome assembly and the previous genome assembly were merged by GAA (v1.1) (Yao et al., 2012). After that, ErrorCorrection and GapCloser modules in SOAPdenovo2 were used sequentially for reads correction and gaps close. Finally, the properly aligned BAC-end sequences (produced before) were used to construct the final scaffolds using an in-house Perl script.

### SSRs and Sequence Variants Identification

The updated genome assembly was used for SSR identification by MISA (v1.0) (Thiel et al., 2003) (**Table 1**). The re-sequencing data of 62 date palm cultivars produced by Hazzouri et al. (2015) were downloaded from the Sequence Read Archive (SRA) of National Center of Biotechnology Information (NCBI) (Supplementary Table S1). The raw data was trimmed by Trimmomatic (v0.33) (Bolger et al., 2014) and aligned to the reference genome using the mem algorithm of BWA (v0.7.12) (Li and Durbin, 2009). The sequence variants were identified by samtools and bcftools (Li and Durbin, 2009; Li, 2011a,b). To reduce the false positives, SNPs presented more than 20% cultivars were kept for subsequent analysis. Primer3 (v3-2.3.7) was used for PCR primer design with default parameters except for “PRIMER_PRODUCT_SIZE_RANGE = 100–280” (Koressaar and Remm, 2007; Untergasser et al., 2012). The putative biological consequences of sequence variants on gene models were predicted by ANNOVAR (v2013-05-20) (Wang et al., 2010).

**Table 1:** Frequency of various types of short sequence repeats (SSRs) in date palm.

Source	Type		Mono		Di		Tri		Tetra		Penta		Hexa	
	Total	Total2a	No.	%	No.	%b	No.	%b	No.	%b	No.	%b	No.	%b
Qatar	–	105183	–	–	52442	49.86	28503	27.10	5555	5.28	12873	12.24	5810	5.52
KACST	287584	118135	169449	–	86033	72.83	23423	19.83	7112	6.02	1028	0.87	539	0.46

*Mono, mononucleotide; Di, dinucleotide; Tri, trinucleotide; Tetra, tetranucleotide; Penta, pentanucleotide; Hexa, hexanucleotide; a, total2 is the total SSRs number for type Di, Tri, Tetra, Penta, and Hexa; b, the percent is corresponding to total2*.

### Phylogenetic Analysis

The phylogenetic tree was constructed using all SNP sites of the varieties by MEGA (NJ method with 1,000 bootstrap, version 6.06) (Tamura et al., 2013). The gaps or missing data were eliminated when the site coverage below 90%. The constructed phylogenetic tree was visualized with EvolView, an online phylogenetic tree visualization tool (Zhang et al., 2012). Principal component analysis (PCA) of SNP genotypes for 62 cultivars was carried out using EIGENSTRAT (Price et al., 2006). The population structure was analyzed using STRUCTURE (Pritchard et al., 2000). The *k*-value of STRUCTURE means the population can be divided into K categories. Each *k*-value was repeated five times. CLUMPP (version 1.1.2) was used to permute the clusters generated from independent STRUCTURE runs (Jakobsson and Rosenberg, 2007).

### Website Construction

The *DRDB* contained both SNPs and SSRs data from 62 date palm cultivars. The website was built on LAMP stack (Linux, Apache, MySQL, and PHP). MySQL was used for data storage on the server side. The front-end interface was implemented with Bootstrap and Angular.

## Results

### The Updated Date Palm Genome Assembly

We re-assembled the palm genome by incorporating newly sequenced HiSeq data (∼130X) produced in our lab and obtained a new genome assembly of 602,484,697 bp in length. As summarized in **Table 2**, comparing the new version (referred as to version 2 below) with the old version (referred as to version 1 below), the number of scaffolds and N bases were reduced to 60.87 and 56.57%, respectively. At contig level, the quality of version 2 was significantly improved; for example, N50 was increased threefold to 34.35 kb, and the number of contigs was decreased ∼50% to 69,963 (**Table 2**). Our results suggested that the new genome assembly could not be further improved by adding more shot-read data with limited insert size (≤8 kb).

**Table 2:** Comparison of the version 1 and version 2 date palm genome assembly.

	Version 1	Version 2 (Added HiSeq)
Assembled genome size (contig size ≥ 500 bp)	558 Mb	543 Mb
Sequencing platform	454, SOLiD	454, SOLiD, HiSeq2000
Scaffold statistics:		
Number	82,354	50,131
Average size (bp)	6,776	10,834
Maximum size (bp)	4,533,682	8,487,612
N bases (bp)	42,166,951 (7.56%)	23,854,181 (4.39%)
N50 (bp)	329,932	349,197
Contig statistics:		
Number	151,198	69,943
Average size (bp)	3,405	7,423
Maximum size (bp)	100,688	302,039
N50 (bp)	10,537	34,351

### SSRs and SNPs

**FIGURE 1:**
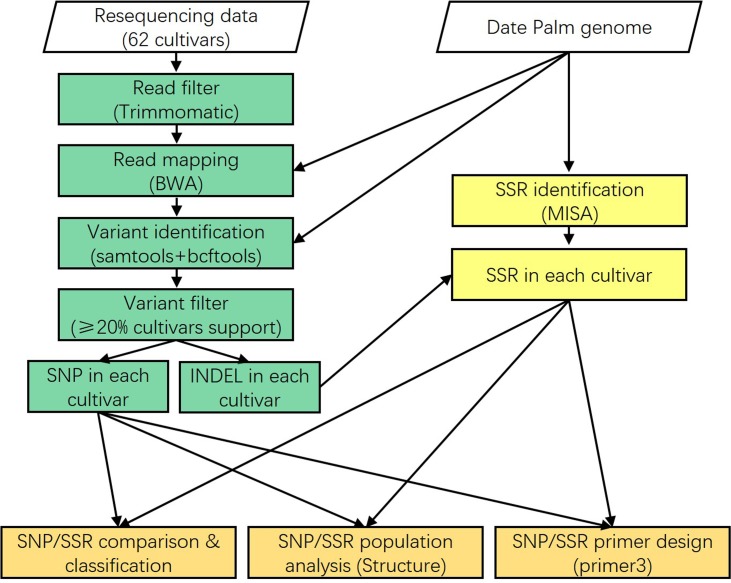
Single nucleotide polymorphisms (SNPs) and short sequence repeats (SSRs) identification pipeline. The 62 date palm cultivars were used to detect SNPs and SSRs. Classification, population structure, and primer design were performed for these SNPs and SSRs.

Using the updated date palm genome sequence, we identified 246,445 SSRs. Among them, 31,544 (12.80%) were compound SSRs (**Figure 1**) and 26,148 (10.61%) were imperfect SSRs. The imperfect SSRs in date palm are significantly less than those in chickpea (17%) (Winter et al., 1999) and lentils (27.1%) (Hamwieh et al., 2009), similar to those in *Elaeis guineensis* (10.87%), and more than those in *Oryza sativa* (9.60%), *Aegilops tauschii* (7.53%), *Zea mays* (5.72%), and *Brachypodium distachyon* (5.47%). Mononucleotide SSRs and dinucleotide SSRs are the most abundant types (58.92 and 29.92%, respectively); among the dinucleotide SSRs, AG/TC (50.50%) is the most abundant, followed by AT/TA (35.50%), AC/TG (13.25%), and CG/GC (0.75%). Trinucleotide SSRs account for 8.14% of the total SSRs, among which AAG/TTC (30.81%) is the most abundant, followed by AAT/TTA (23.20%) and AGG/TCC (17.97%). Using Primer 3, we were able to design high-quality primer pairs for 174,497 (70.81%) SSRs.

For the published SSRs that we examined, most of them were covered by our dataset. For example, the SSRs that we inferred here contained 9 of 16 ones proposed previously Billotte et al. (2004) and 11 of 17 SSR loci Akkak et al. (2009); we also found 8 of 19 SSR primers Hamwieh et al. (2010) and 13 of 30 SSR primers by Elmeer et al. (2011) in our data (Supplementary Table S2).

We identified 6,375,806 SNPs (raw read depth≥3 in 90% cultivars) among 62 date palm cultivars. To reduce false positives, we only kept those (5,572,650, 87.40%) found in more than 20% cultivars for downstream analysis. We designed high-quality PCR primers for 4,177,778 (65.53%) SNPs.

These genetic variants and pre-designed PCR primers could be a valuable resource for researchers for genetic and breeding studies.

### Population Structure

**FIGURE 2:**
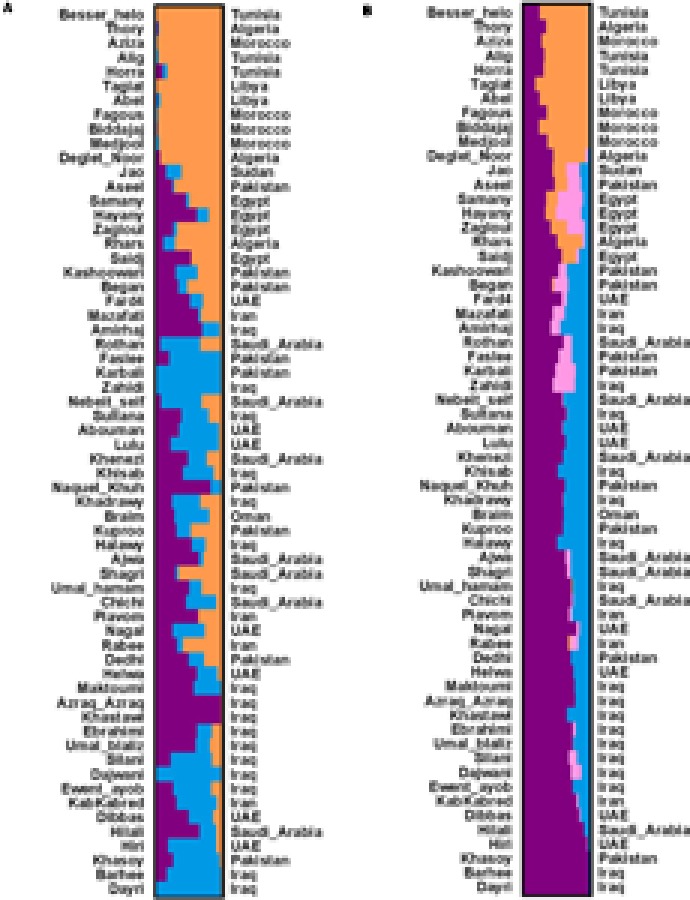
**(A)** Population structure based on SNPs (*k* = 3). The 62 date palm cultivars were grouped into three clusters. The color bar represents the component of ancestral origin. **(B)** Population structure based on SSRs (*k* = 4). The 62 date palm cultivars were grouped into three clusters. The color bar represents the component of ancestral origin. The two clusters (Middle East and South Asia) are nearly same according to the geography locations among four SSR-based clusters. Thus, the SSR-based clusters are considered as three clusters.

**FIGURE 3:**
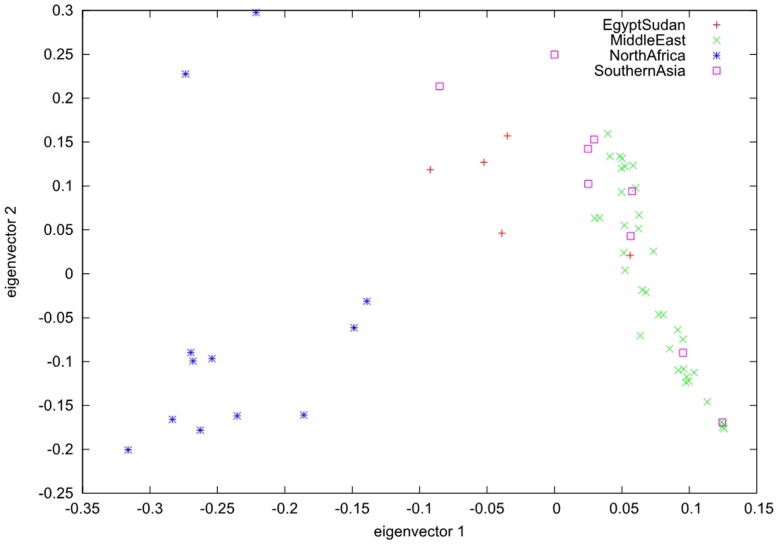
Principal component analysis (PCA) analyses of SNPs shows three distinct clusters of date palm cultivars. The scatter plot shows the PCA analysis of SNPs of 62 date palm cultivars. Each shape of dot represents one distinct geographic location. The plot shows three distinct clusters.

To elucidate the population genetic structure, we selected 34,346 SSRs with length polymorphism and 57,310 SNPs in the largest scaffold of data palm genome for further analysis with STRUCTURE. We run STRUCTURE with *k* = 1–7 for SSRs and *k* = 1–6 for SNPs; we run STRUCTURE HARVESTER program to determine the best *k*-values for SSRs (*k* = 4) and SNPs (*k* = 3) (**Supplementary Figure S1**) (Earl, 2012). Our results showed that date palm can be divided into three genetically differentiated clusters, North Africa, Egypt – Sudan and Middle East – South Asian, with Egypt – Sudan being the admixture of North Africa and Middle East – South Asian cultivars (**Figure 2**). We further confirmed the clustering using PCA using all SNPs (**Figure 3**).

### Construction of the Online Database

#### The Web Interface

**FIGURE 4:**
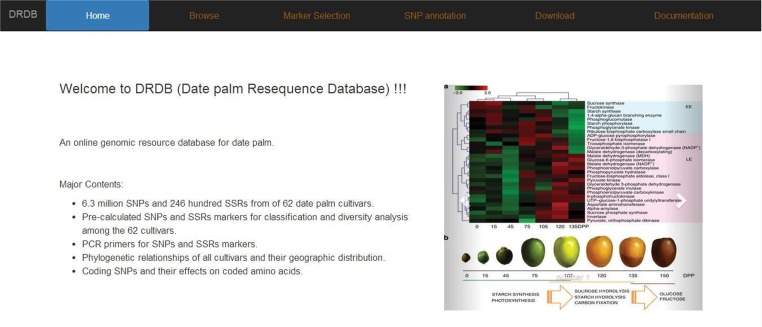
Date palm genome database (DRDB) home page.

We designed *DRDB* with a user-friendly web interface, incorporated visualization tools for genomic features and provided powerful searching functionality. *DRDB* comes with several pages including Home, Browse, Marker selection, SNP annotation, Download, and Documentation. Below we introduce selected pages in details (**Figure 4**).

#### The Browse Page

**FIGURE 5:**
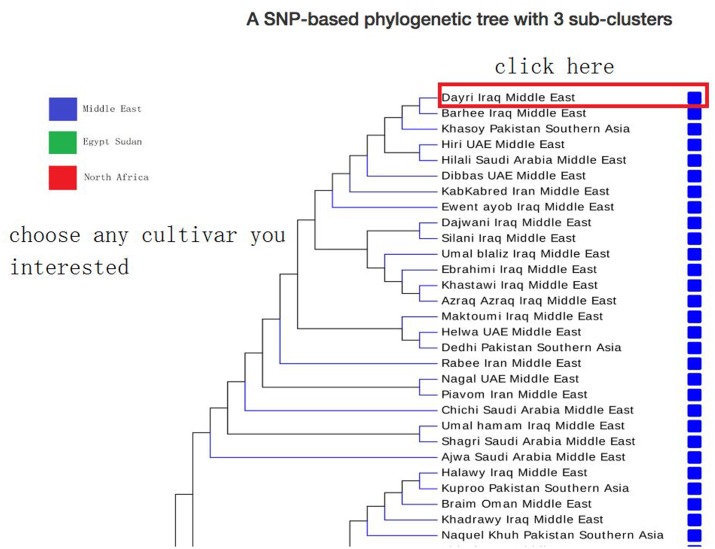
Date palm genome database browse page. A SNP-based phylogenetic tree was shown in browse page. Users can choose any cultivar they interested and then jump to the next level page to view SNP/SSR detailed information.

**FIGURE 6:**
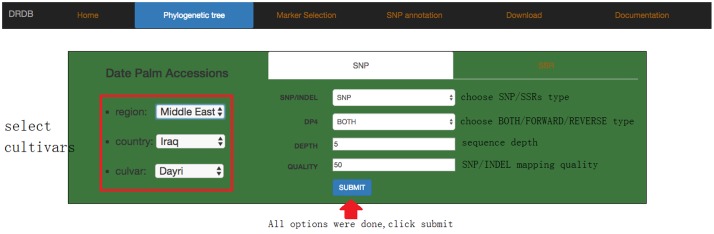
Date palm genome database cultivar information page. The next level page of browse is cultivar information page. Users can select any interested cultivar and marker type (SNP or SSR).

In browse page, the phylogenetic tree that was derived from the SNP data is shown; the topology of this tree suggests that there are at least three sub-groups of date palm cultivars, each features distinct geographic locations: Middle East, Egypt Sudan, and North Africa. Users can click on any cultivar they interested in, and view a list of its available SNPs and SSRs in a new page (**Figure 5**), which lists all available SNPs and SSRs for user-selected cultivars. Users can search for SNPs and SSRs they are interested by changing different parameters (such as “DEPTH,” “QUALITY” in SNP data or “SSR type,” “Product size” in SSR data) (**Figure 6**).

#### The Marker Selection Page

**FIGURE 7:**
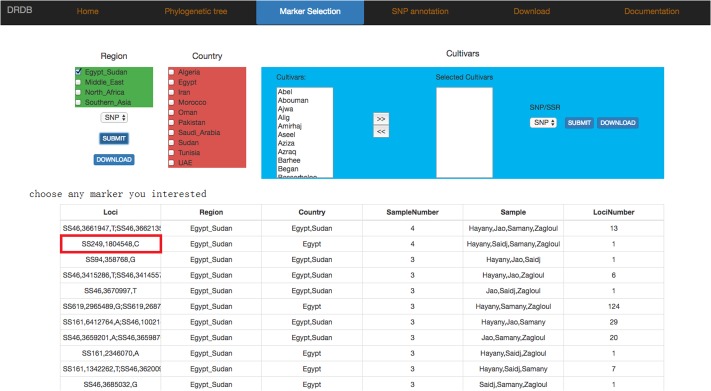
Date palm genome database marker selection page. Marker selection page provides SNP/SSR markers of different cultivars. Users can pick any marker to view marker frequency and detailed information in the next level page.

**FIGURE 8:**
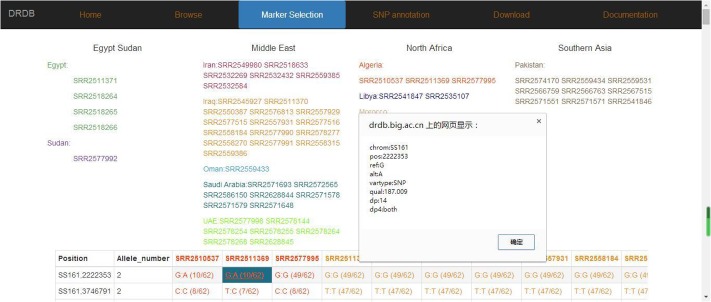
Date palm genome database marker frequency page. Marker frequency and detail information are shown in this page.

In marker selection page, we divided SNP and SSR markers into three levels (Region, Country, and Cultivars). Users can select any combination of maker types (SNP or SSR) and levels (Region, Country or Cultivars) they are interested, and view the results in a new page (**Figure 7**), which contains also the allele frequency and detailed information of selected markers, including “position, ref, alt, dp, qual” in SNP marker or “SSR_type, size, type, cov” in SSR marker (**Figure 8**).

#### Other Pages

In addition to “Browse” and “Marker selection” pages, we also developed “SNP annotation” page. Users can search by the names of SNPs or their putative functional consequences such as “non-synonymous SNV,” “synonymous SNV,” “frameshift substitution,” and “non-frameshift substitution.” We also included gene annotation from external data sources including UNIPROT. All the SNPs, SSRs and SNP effect data can be downloaded in “Download” page. The “Documentation” page shows the detail introductions of *DRDB*.

## Discussion

Date palm genome database provides users with a comprehensive genomic resources for date palm. The updated date palm genome assembly reduces the numbers of scaffolds and ambiguous bases significantly (60.87 and 56.57%) comparing with the previous genome assembly. Large (≥20 kb) insert size sequencing data, genetic map and physical map are needed for improving genome assembly in the future.

Date palm has unique genetic diversity due to long-lived life cycle, vegetative propagation, and complex origin. Study data palm genetic diversity and classification is an important research aspect. Hamwieh et al. (2010) identified previously ∼1000 SSR markers in an early version of the draft genome with genome size about 50% of our current assembly; they did not consider the SSRs variability among date palm cultivars, nor provided any SSR markers. Hazzouri et al. (2015) identified SNPs in 62 date palm cultivars, but they didn’t provide convenient tools for SNPs comparison nor designed primers for maker development.

In this study, based on an improved genome assembly, we identified 246,445 SSRs and 6,375,806 SNPs in 62 cultivars and designed high-quality PCR primers for most of variations (70.81% in SSRs and 65.53% in SNPs). These genetic variants and pre-designed PCR primers will be a valuable resource for researchers to perform genetic and breeding studies.

The origin of date palm is controversial because date palm cultivation spread out many countries starting in ancient times. Based on SSRs and SNPs, we elucidated the population genetic structure of date palm and the results showed that date palm can be divided into three genetically differentiated clusters, North Africa, Egypt – Sudan and Middle East – South Asian, with Egypt – Sudan being the admixture of North Africa and Middle East – South Asian cultivars.

To facilitate the use of our data, we developed DRDB for distinguishing the different sub-types of date palms and mining the genetic variation of interest. DRDB provides visualization tools for genomic features and powerful searching functionality. Users can download all these data from download page.

In summary, DRDB provided an updated date palm genome assembly and enabled genetic variation searching for 62 date palm cultivars rapidly and conveniently, which sets the stage for further genetic and breeding studies.

## Author Contributions

ZH, CZ, and WL contributed equally to this work. SH, W-HC, and HA led the research. ZH and CZ constructed the database. WL, QL, and TW analyzed the data. ZH, W-HC, and WL wrote and revised the manuscript.

## Conflict of Interest Statement

The authors declare that the research was conducted in the absence of any commercial or financial relationships that could be construed as a potential conflict of interest.

## Supplementary Material

FIGURE S1 (A) *K*-value selection of population structure for SNP. The picture of SNP-based *k*-value selection shows that 3 is the proper *k*-value, which is the highest value of the *y*-axis. **(B)** *K*-value selection of population structure for SSR. The picture of SSR-based *k*-value selection shows that 4 is the proper *k*-value, which is the highest value of the *y*-axis.
