# Supplementary figures and images for: DRDB: An Online Date Palm Genomic Resource Database
Source: Front Plant Sci. 2017 Nov 2;8:1889. doi: 10.3389/fpls.2017.01889 (PMC5701633; doi:10.3389/fpls.2017.01889)

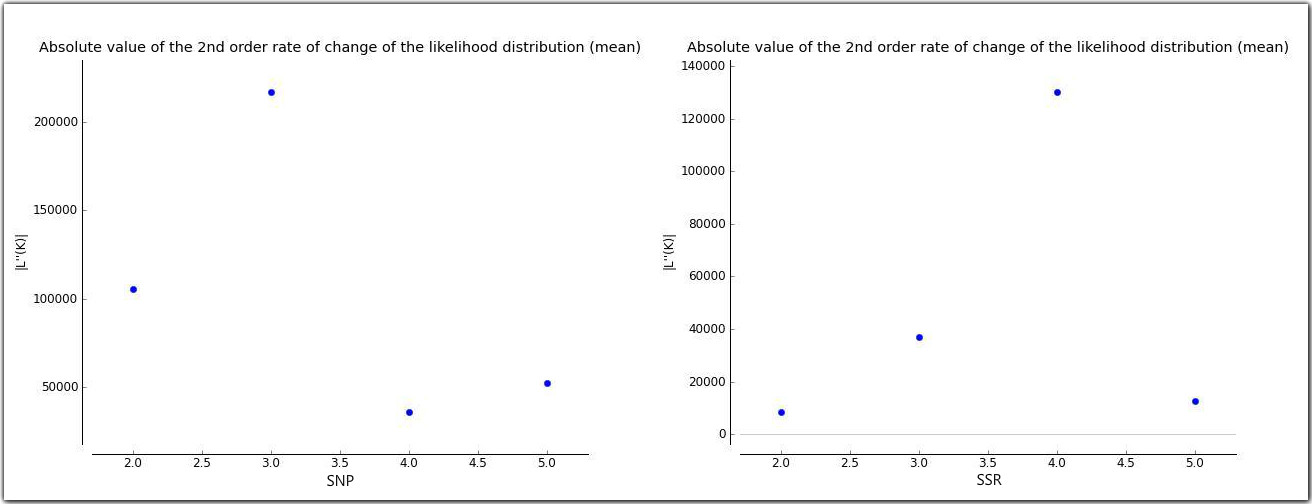

Supplement: FIGURE S1 — (A) K-value selection of population structure for SNP. The picture of SNP-based k-value selection shows that 3 is the proper k-value, which is the highest value of the y-axis. (B) K-value selection of population structure for SSR. The picture of SSR-based k-value selection shows that 4 is the proper k-value, which is the highest value of the y-axis. [file Image_1.jpg]
